# *De novo* genome assembly of *Camptotheca acuminata*, a natural source of the anti-cancer compound camptothecin

**DOI:** 10.1093/gigascience/gix065

**Published:** 2017-07-24

**Authors:** Dongyan Zhao, John P. Hamilton, Gina M. Pham, Emily Crisovan, Krystle Wiegert-Rininger, Brieanne Vaillancourt, Dean DellaPenna, C. Robin Buell

**Affiliations:** 1 Department of Plant Biology, Michigan State University, 612 Wilson Rd., East Lansing, MI 48824, USA;; 2 Department of Biochemistry and Molecular Biology, Michigan State University, 603 Wilson Rd, Rm212, East Lansing, MI 48824, USA

**Keywords:** *Camptotheca acuminata*, camptothecin, genome assembly, genome annotation, tandem duplications

## Abstract

*Camptotheca acuminata* is 1 of a limited number of species that produce camptothecin, a pentacyclic quinoline alkaloid with anti-cancer activity due to its ability to inhibit DNA topoisomerase. While transcriptome studies have been performed previously with various camptothecin-producing species, no genome sequence for a camptothecin-producing species is available to date. We generated a high-quality *de novo* genome assembly for *C. acuminata* representing 403 174 860 bp on 1394 scaffolds with an N50 scaffold size of 1752 kbp. Quality assessments of the assembly revealed robust representation of the genome sequence including genic regions. Using a novel genome annotation method, we annotated 31 825 genes encoding 40 332 gene models. Based on sequence identity and orthology with validated genes from *Catharanthus roseus* as well as Pfam searches, we identified candidate orthologs for genes potentially involved in camptothecin biosynthesis. Extensive gene duplication including tandem duplication was widespread in the *C. acuminata* genome, with 2571 genes belonging to 997 tandem duplicated gene clusters. To our knowledge, this is the first genome sequence for a camptothecin-producing species, and access to the *C. acuminata* genome will permit not only discovery of genes encoding the camptothecin biosynthetic pathway but also reagents that can be used for heterologous expression of camptothecin and camptothecin analogs with novel pharmaceutical applications.

## Data Description

### Background information on camptothecin, a key anti-cancer natural product

**Figure 1:**
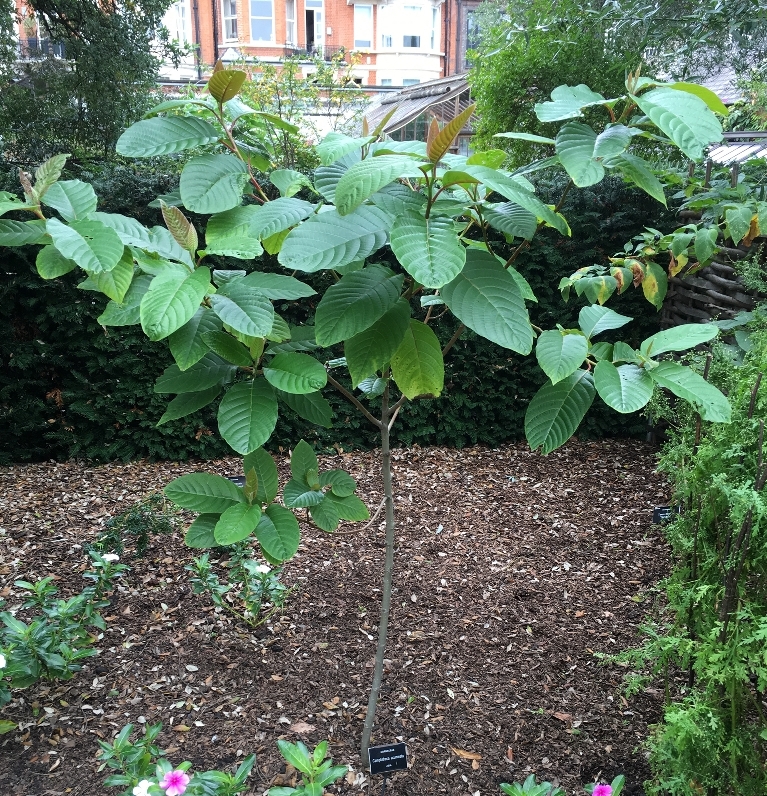
*Camptotheca acuminata* Decne, the Chinese Happy Tree, is a member in the Nyssaceae family that produces the anticancer compound camptothecin.

**Figure 2:**
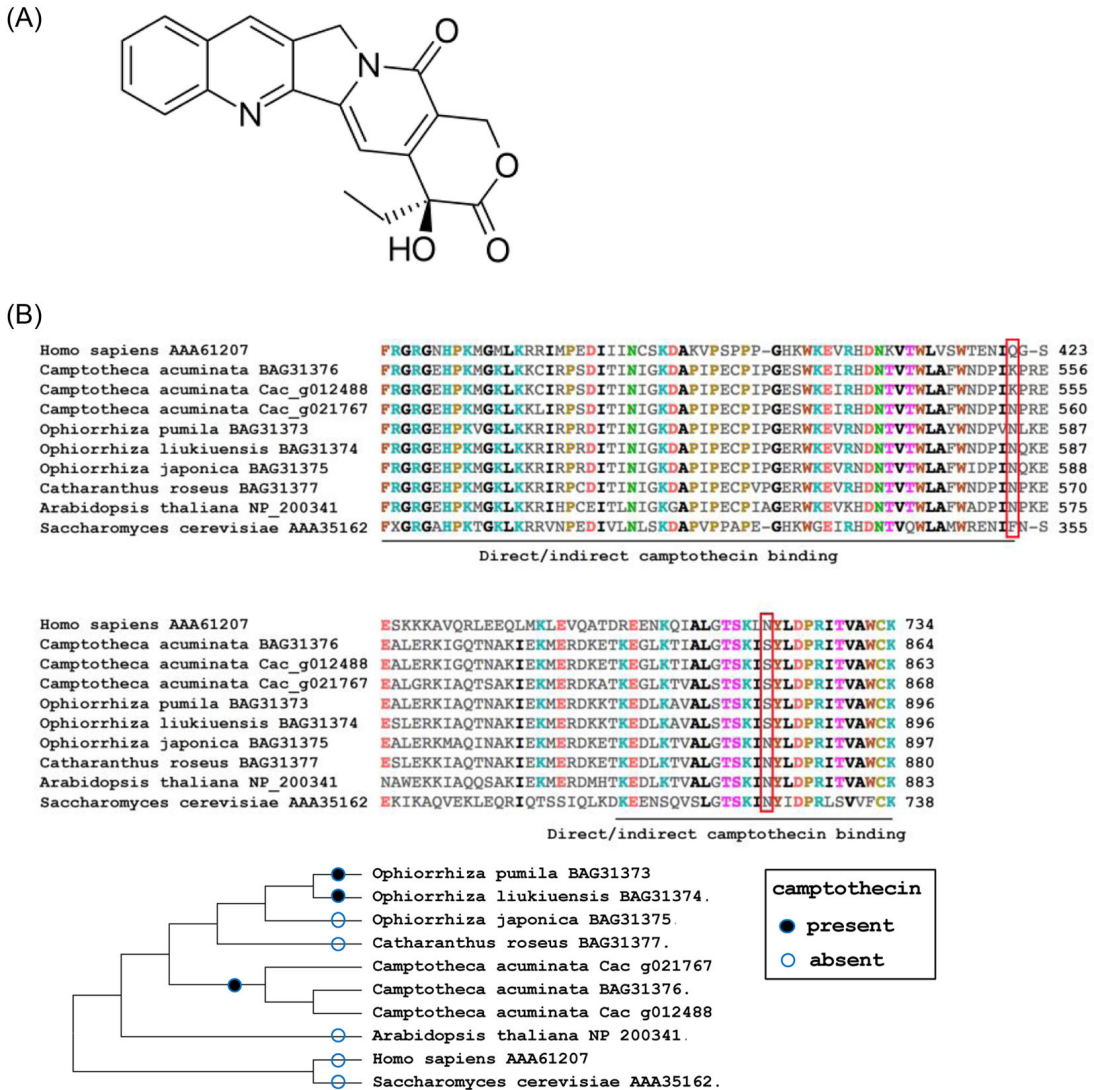
Genome aspects of *Camptotheca acuminata*. (**A**) Structure of camptothecin. (**B**) Key amino acid mutations (red rectangles) in DNA topoisomerase I in camptothecin-producing and non-producing species and their phylogenetic relationship.

*Camptotheca acuminata* Decne, also known as the Chinese Happy Tree (Fig. 1), is a eudicot asterid Cornales tropical tree species within the Nyssaceae family [1] that also contains *Nyssa* spp (tupelo) and *Davidia involucrate* (dove tree); no genome sequence is available for any member of this family. *C. acuminata* is 1 of a limited number of plant species that produce camptothecin, a pentacyclic quinoline alkaloid (Fig. 2A) with anti-cancer activity due to its ability to inhibit DNA topoisomerase [2]. Due to poor solubility, derivatives such as irinotecan and topotecan, rather than camptothecin, are currently in use as approved cancer drugs. The significance of these derivatives as therapeutics is highlighted by the listing of irinotecan on the World Health Organization Model List of Essential Medicines [3]. While transcriptome studies have been performed previously with various camptothecin-producing species including *C. acuminata* and *Ophiorrhiza pumila* (e.g., [4–6]), no genome sequence for a camptothecin-producing species is available to date. We report on the assembly and annotation of the *C. acuminata* genome, the characterization of genes implicated in camptothecin biosynthesis, and highlight the extent of gene duplication that provides new templates for gene diversification.

### RNA isolation, library construction, sequencing, and transcriptome assembly

Transcriptome assemblies were constructed using 9 developmental RNA-sequencing (RNA-seq) datasets, described in a previous study [4], that included immature bark, cotyledons, immature flower, immature fruit, mature fruit, mature leaf, root, upper stem, and lower stem. Adapters and low-quality nucleotides were removed from the RNA-seq reads using Cutadapt v. 1.8 (Cutadapt, RRID:SCR_011841) [7], and contaminating ribosomal RNA reads were removed. Cleaned reads from all 9 libraries were assembled using Trinity v. 20140717 (Trinity, RRID:SCR_013048) [8] with a normalization factor of ×50 using default parameters. Contaminant transcripts (5669 total) were identified by searching the *de novo* transcriptome assembly against the National Center for Biotechnology Information (NCBI) non-redundant nucleotide database using BLAST+ (v. 2.2.30) [9, 10] with an E-value cutoff of 1e-5; transcripts whose best hits were a non-plant sequence were removed from the transcriptome.

For additional transcript support for use in a genome-guided transcriptome assembly to support genome annotation, strand-specific RNA-seq reads were generated by isolating RNA from root tissues and sequencing of Kappa TruSeq Stranded libraries on an Illumina HiSeq 2500 platform generating 150 nt paired-end reads (BioSample ID: SAMN06229771). Root RNA-seq reads were assessed for quality using FASTQC v. 0.11.2 (FASTQC, RRID:SCR_014583) [11] using default parameters and were cleaned as described above.

### DNA isolation, library construction, and sequencing

The genome size of *C. acuminata* was estimated at 516 Mb using flow cytometry, suitable for *de novo* assembly using the Illumina platform. DNA was extracted from young leaves of *C. acuminata* at the vegetative growth stage using CTAB [12]. Multiple Illumina-compatible paired-end libraries (Table 1) with insert sizes ranging from 180–609 bp were constructed as described previously [13] and sequenced to 150 nt in paired-end mode on an Illumina HiSeq2000. Mate-pair libraries (Table 1) with size ranges of 1.3–8.9 kb were made using the Nextera Kit (Illumina, San Diego, CA, USA), as per the manufacturer's instructions, and sequenced to 150 nt in paired-end mode on an Illumina HiSeq2000.

**Table 1:** Input libraries and sequences for *de novo* assembly of the *Camptotheca acuminata* genome

BioProject ID	BioSample ID	Fragment size (bp)	No. of cleaned read pairs	Use
Paired end				
PRJNA361128	SAMN06220985	180	96 955 546	ALLPATHS-LG assembly
PRJNA361128	SAMN06220986	268	89 381 055	ALLPATHS-LG assembly
PRJNA361128	SAMN06220987	352	61 207 691	GapCloser
PRJNA361128	SAMN06220988	429	50 688 562	GapCloser
PRJNA361128	SAMN06220989	585	21 856 610	GapCloser
PRJNA361128	SAMN06220990	609	22 217 954	GapCloser
Mate pair				
PRJNA361128	SAMN06220991	8111	9 923 643	ALLPATHS-LG assembly
PRJNA361128	SAMN06220992	7911	7 652 519	ALLPATHS-LG assembly
PRJNA361128	SAMN06220993	1377	12 800 554	ALLPATHS-LG assembly
PRJNA361128	SAMN06220994	3179	13 138 503	ALLPATHS-LG assembly
PRJNA361128	SAMN06220995	8879	13 599 241	ALLPATHS-LG assembly

All libraries were sequenced in paired-end mode, generating 150 nt reads.

### Genome assembly

Paired-end reads (Table 1) were assessed for quality using FASTQC v. 0.11.2 (FASTQC, RRID:SCR_014583) [11] using default parameters and cleaned for adapters and low-quality sequences using Cutadapt v. 1.8 (Cutadapt, RRID:SCR_011841) [7], and only reads in pairs with each read ≥25 nt were retained for genome assembly. Mate pair libraries (Table 1) were processed using NextClip v. 1.3.1 (NextClip, RRID:SCR_005465) [14], and only reads from Categories A, B, and C were used for the assembly. Using ALLPATHS-LG v. 44837 (ALLPATHS-LG, RRID:SCR_010742) [15] with default parameters, 2 paired-end read libraries (180 and 268 bp insert libraries) and all 5 mate pair libraries (Table 1) were used to generate an initial assembly of 403.2 Mb with an N50 contig size of 108 kbp and an N50 scaffold size of 1752 kbp (Tables 1 and 2). Gaps (5076) in this initial assembly were filled using SOAP GapCloser v. 1.12r6 (GapCloser, RRID:SCR_015026) [16] with 4 independent paired-end libraries (352, 429, 585, and 609 bp inserts) (Table 1); 12 468 362 bp of the estimated 16 471 841 bp of gaps was filled, leaving a total of 3825 gaps (3772,191 Ns). The assembly was checked for contaminant sequences based on alignments to the NCBI non-redundant nucleotide database using BLASTN (E-value = 1e-5) [10]. A single scaffold of 5156 bp that matched a bacterium sequence with 100% coverage and 100% identity was removed. Subsequently, 5 scaffolds of less than 1 kbp were removed, resulting in the final assembly of 403 174 860 bp, comprised of 1394 scaffolds with an N50 scaffold size of 1752 kbp (Tables 1 and 2) and 0.9% Ns.

**Table 2:** Metrics of the final assembly of *Camptotheca acuminata* genome

Metric	Value
Total scaffold length (bp)	403 174 860
Total no. of scaffolds (bp)	1394
Maximum scaffold length (bp)	8 423 530
Minimum scaffold length (bp)	1002
N50 scaffold size (bp)	1 751 747
N50 contig size (bp)	107 594
No. of Ns	3 772 191 (0.9%)
No. gaps	3825

Quality assessments revealed a robust high-quality assembly, with 98% of the paired-end genomic sequencing reads aligning to the assembly, of which 99.97% aligned concordantly. With respect to genic representation, 95.3% of RNA-seq-derived transcript assemblies [4] and 74 119 of 74 682 (99%) pyrosequencing transcript reads from a separate study [5] aligned to the genome assembly. A total of 93.6% of conserved Embryophyta BUSCO (BUSCO, RRID:SCR_015008) proteins were present in the assembly as full-length sequences, with an additional 2.5% of the Embryophyta proteins fragmented [17].

### Genome annotation

We used a novel genome annotation method to generate high-quality annotation of the *C. acuminata* genome, in which we repeat-masked the genome, trained an *ab initio* gene finder with a genome-guided transcript assembly, and then refined the gene models using additional genome-guided transcript assembly evidence to generate a high-quality gene model set. We first created a *C. acuminata* specific custom repeat library (CRL) using MITE-Hunter v. 2011 [18] and RepeatModeler v. 1.0.8 (RepeatModeler, RRID:SCR_015027) [19]. Protein coding genes were removed from each repeat library using ProtExcluder.pl v. 1.1 [20] and combined into a single CRL, which hard-masked 143.6 Mb (35.6%) of the assembly as repetitive sequence using RepeatMasker v. 4.0.6 (RepeatMasker, RRID:SCR_012954) [21]. Cleaned root RNA-seq reads (Table S1, BioSample ID: SAMN06229771) were aligned to the genome assembly using TopHat2 v. 2.0.13 (TopHat, RRID:SCR_013035) [22] in strand-specific mode with a minimum intron length of 20 bp and a maximum intron length of 20 kb; the alignments were then used to create a genome-guided transcriptome assembly using Trinity v. 2.2.0 (Trinity, RRID:SCR_013048) [23]. The RNA-seq alignments were used to train AUGUSTUS v. 3.1 (Augustus: Gene Prediction, RRID:SCR_008417) [24], and gene predictions were generated with AUGUSTUS [25] using the hard-masked assembly. Gene model structures were refined by incorporating evidence from the genome-guided transcriptome assembly using PASA2 v. 2.0.2 (PASA, RRID:SCR_014656) [26, 27] with the parameters MIN_PERCENT_ALIGNED = 90 and MIN_AVG_PER_ID = 99. After annotation comparison, models that PASA identified as being merged and a subset of candidate camptothecin biosynthetic pathway genes identified as mis-annotated were manually curated. The final high-confidence gene model set consists of 31 825 genes encoding 40 332 gene models. Functional annotation was assigned using a custom pipeline using WU-BLASTP [28] searches against the *Arabidopsis thaliana* annotation (TAIR10) [29] and Swiss-Prot plant proteins (downloaded on 17 August 2015), and a search against Pfam (v. 29) using HMMER v. 3.1b2 (Hmmer, RRID:SCR_005305) [30]. This resulted in 34 143 gene models assigned a putative function, 2011 annotated as conserved hypothetical, and 4178 annotated as hypothetical.

*C. acuminata* is insensitive to camptothecin due to mutations within its own DNA topoisomerase [31], and we identified 2 topoisomerase genes in our annotated gene set, 1 of which matches the published *C. acuminata* topoisomerase (99.78% identity, 100% coverage) and includes the 2 mutations that confer resistance to camptothecin (Fig. 2B), 1 mutation is specific to *C. acuminata*, and the other is present in both *C. acuminata* and 2 camptothecin-producing *Ophiorrhiza* species. Further quality assessments of our annotation with 35 nuclear-encoded *C. acuminata* genes available from GenBank revealed an average identity of 99.5% with 100% coverage in our annotated proteome while a single gene encoding 1-deoxy-D-xylulose 5-phosphate reductoisomerase (ABC86579.1) had 88.2% identity with 100% coverage, which may be attributable to differences in genotypes. One mRNA reported to encode a putative strictosidine beta-D-glucosidase (AES93119.1) was found to have a retained intron that, when removed, aligned with 99.3% identity yet reduced coverage (66%) as it was located at the end of a short scaffold. Collectively, the concordant alignment of whole-genome shotgun sequence reads to the assembly, the high representation of genic regions as assessed by independent transcriptome datasets (RNA-seq and pyrosequencing), and the core Embryophyta BUSCO proteins, when coupled with the high-quality gene models as revealed through alignments with cloned *C. acuminata* genes, indicate that we have generated not only a high-quality genome assembly for *C. acuminata* but also a robust set of annotated gene models.

### Gene duplication and orthology analyses

During our annotation efforts, it was readily apparent that there was substantial gene duplication, including tandem gene duplication in the *C. acuminata* genome. Paralogous clustering of the *C. acuminata* proteome revealed 5516 paralogous groups containing 15 806 genes. We identified tandem gene duplications in the *C. acuminata* genome based on if (i) 2 or more *C. acuminata* genes were present within an orthologous/paralogous group; (ii) there were no more than 10 genes in between on a single scaffold; and (iii) the pairwise gene distance was less than 100 kbp [32]. Under these criteria, 2571 genes belonging to 997 tandem duplicated gene clusters were identified. Gene ontology analysis showed that tandem duplicated genes are significantly enriched in “response to stress” (*P* < 0.0001, *χ*^2^ test) while they are under-represented in most other processes, especially “other cellular processes” and “cell organization and biogenesis” (*P* < 0.0001, *χ*^2^ test).

**Figure 3:**
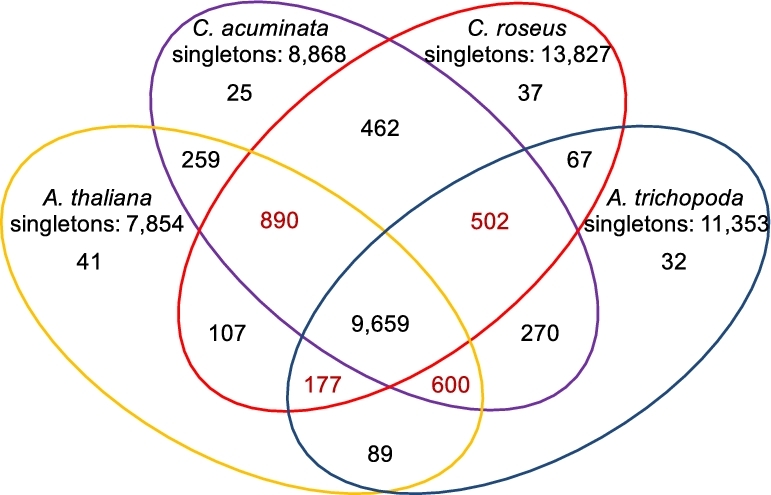
Venn diagram showing orthologous and paralogous groups between *Amborella trichopoda, Arabidopsis thaliana, Camptotheca acuminata*, and *Catharanthus roseus.*

To our knowledge, *C. acuminata* is the first species within the Nyssaceae family with a genome sequence. To better understand the evolutionary relationship of *C. acuminata* with other asterids and angiosperms, we identified orthologous and paralogous groups using our annotated *C. acuminata* proteome and the proteomes of 3 other key species (*Arabidopsis thaliana, Amborella trichopoda*, and *Catharanthus roseus*) using OrthoFinder (v. 0.7.1) [33] with default parameters. A total of 12 667 orthologous groups containing at least a single *C. acuminata* protein were identified, with 9659 orthologous groups common to all 4 species (Fig. 3; Table S2). Interestingly, *C. acuminata* contains fewer singleton genes (8868) than *A. trichopoda* and *C. roseus*, and gene ontology analysis demonstrated that these genes were highly enriched in “transport,” “response to stress,” and “other metabolic and biological processes” (*P* < 0.0001, *χ*^2^ test) while they were dramatically under-represented in “unknown biological processes” (*P* < 0.0001, *χ*^2^ test), suggesting that these genes may be involved in stress responses and other processes specific to *C. acuminata*.

### Uses for the *C. acuminata* genome sequence and annotation

**Figure 4:**
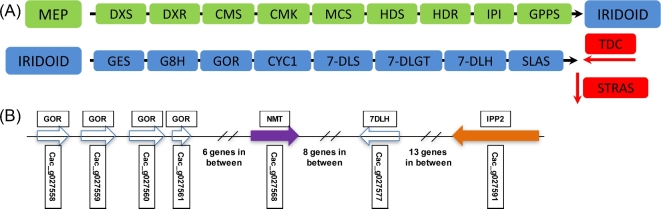
Key portions of the proposed camptothecin biosynthetic pathway and an example of physical clustering of candidate genes in *Camptotheca acuminata*. (**A**) The methylerythritol phosphate (MEP) pathway (green), iridoid pathway (blue), and condensation of secologanic acid with tryptamine via strictosidinic acid synthase (STRAS) to form strictosidinic acid prior to downstream dehydration, reduction, and oxidation steps yielding camptothecin. 7-DLGT: 7-deoxyloganetic acid glucosyltransferase; 7-DLH: 7-deoxyloganic acid hydroxylase; 7-DLS: 7-deoxyloganetic acid synthase; CMK: 4-diphosphocytidyl-2-C-methyl-D-erythritol kinase; CMS: 4-diphosphocytidyl-methylerythritol 2-phosphate synthase; CYC1: iridoid cyclase 1; DXR: 1-deoxy-D-xylulose-5-phosphate reductoisomerase; DXS: 1-deoxy-D-xylulose 5-phosphate synthase 2; G8H: geraniol 8-hydroxylase; GES: plastid geraniol synthase; GOR: 8-hydroxygeraniol oxidoreductase; GPPS: geranyl pyrophosphate synthase; HDR: 1-hydroxy-2-methyl-butenyl 4-diphosphate reductase; HDS: GCPE protein; IPI: plastid isopentenyl pyrophosphate, dimethylallyl pyrophosphate isomerase; MCS: 2C-methyl-D-erythritol 2,4-cyclodiphosphate synthase; SLAS: secologanic acid synthase; TDC: tryptophan decarboxylase. (**B**) Physical clustering of homologs of genes involved in the methylerythritol phosphate, iridoid, and alkaloid biosynthetic pathways of *Catharanthus roseus* on scaffold 151 of *C. acuminata*. Gene IDs are below the arrows. 7DLH: 7-deoxyloganic acid 7-hydroxylase; GOR: 8-hydroxygeraniol oxidoreductase; IPP2: isopentenyl diphosphate isomerase II; NMT: 16-hydroxy-2,3-dihydro-3-hydroxytabersonine N-methyltransferase.

Generation of a high-quality genome sequence and annotation dataset for *C. acuminata* will facilitate discovery of genes encoding camptothecin biosynthesis as physical clustering can be combined with sequence similarity and co-expression data to identify candidate genes, an approach that has been extremely useful in identifying genes in specialized metabolism in a number of plant species (see [34–36]). In *C. acuminata*, geranylgeranyl diphosphate from the 2-*C*-methyl-D-erythritol 4-phosphate/1-deoxy-D-xylulose 5-phosphate (MEP) pathway is used to generate secologanic acid via the iridoid pathway; it and tryptamine from tryptophan decarboxylase are condensed by strictosidinic acid synthase to generate strictosidinic acid, which is then converted into camptothecin in the alkaloid pathway via a set of unknown steps (Fig. 4A) [37]. *Catharanthus roseus*, Madagascar periwinkle, produces vinblastine and vincristine via the MEP and iridoid pathways, for which all genes leading to the biosynthesis of the iridoid secologanin have been characterized [35]. Using sequence identity and coverage with characterized *C. roseus* genes from the MEP and iridoid pathways (Fig. 4A), we were able to identify candidate genes for all steps in the MEP and iridoid pathway in *C. acuminata* (Table 3). The downstream steps in camptothecin biosynthesis subsequent to formation of strictosidinic acid involve a broad set of enzymes responsible for reduction and oxidation [37], and a total of 343 cytochrome P450s (56 paralogous gene clusters and 120 singletons) (Table S3) were identified that can serve as candidates for the later steps in camptothecin biosynthesis.

**Table 3:** Identification of candidate camptothecin biosynthetic pathway genes in the *Camptotheca acuminata* genome as revealed by sequence identity and coverage with characterized genes from the 2-*C*-methyl-D-erythritol 4-phosphate/1-deoxy-D-xylulose 5-phosphate and iridoid biosynthetic pathways from *Catharanthus roseus*

Description	Abbreviation	Protein	Camptotheca gene ID	% coverage	% identity
MEP					
1-deoxy-D-xylulose 5-phosphate synthase 2	DXS	ABI35993.1	Cac_g024944.t1	98	77.60
1-deoxy-D-xylulose-5-phosphate reductoisomerase	DXR	AAF65154.1	Cac_g016318.t1	100	88.82
4-diphosphocytidyl-methylerythritol 2-phosphate synthase	CMS	ACI16377.1	Cac_g018722.t1	88	77.82
4-diphosphocytidyl-2-C-methyl-D-erythritol kinase	CMK	ABI35992.1	Cac_g021688.t1	99	76.17
2C-methyl-D-erythritol 2,4-cyclodiphosphate synthase	MCS	AAF65155.1	Cac_g008169.t1	100	73.77
GCPE protein	HDS	AAO24774.1	Cac_g022763.t1	100	88.65
1-hydroxy-2-methyl-butenyl 4-diphosphate reductase	HDR	ABI30631.1	Cac_g014659.t1	100	83.77
Plastid isopentenyl pyrophosphate: dimethylallyl pyrophosphate isomerase	IPI	ABW98669.1	Cac_g008847.t1	76	91.06
Geranyl pyrophosphate synthase	GPPS	ACC77966.1	Cac_g026508.t1	51	76.50
Iridoid					
Geraniol 8-hydroxylase	G8H	CAC80883.1	Cac_g017987.t1	95	76.71
8-hydroxygeraniol oxidoreductase	GOR	AHK60836.1	Cac_g027560.t1	100	71.69
Iridoid synthase	ISY	AFW98981.1	Cac_g006027.t1	100	65.65
Iridoid oxidase	IO	AHK60833.1	Cac_g032709.t1	97	78.44
UDP-glucose iridoid glucosyltransferase	7DLGT	BAO01109.1	Cac_g008744.t1	100	77.11
7-deoxyloganic acid 7-hydroxylase	7DLH	AGX93062.1	Cac_g012663.t1	96	69.58
Loganic acid methyltransferase	LAMT	ABW38009.1	Cac_g005179.t1	95	53.91
Secologanin synthase	SLS	AAA33106.1	Cac_g012666.t1	99	64.94

Only the top hit from the BLAST search is presented.

Though not absolute, physical clustering of genes involved in specialized metabolism has been observed in a number of species across a number of classes of specialized metabolites [34, 38]. With an N50 scaffold size of 1752 kbp, we observed several instances of physical clustering of genes with homology to genes involved in monoterpene indole alkaloid biosynthesis, which may produce related compounds in *C. acuminata*. Using characterized genes involved in the biosynthesis of vinblastine and vincristine from *C. roseus* as queries (Fig. 4A, Table 3) [35], we identified a single *C. acuminata* scaffold (907 kbp, 86 genes) (Fig. 4B) that encoded genes with sequence identity to isopentenyl diphosphate isomerase II within the MEP pathway, 8-hydroxygeraniol oxidoreductase (GOR, 3 complete and 1 partial paralogs), 7-deoxyloganic acid 7-hydroxylase (7DLH) within the iridoid pathway, and a protein with homology to *C. roseus* 16-hydroxy-2,3-dihydro-3-hydroxytabersonine N-methyltransferase (NMT) within the alkaloid pathway, suggesting that access to a high-contiguity genome assembly may facilitate discovery of genes involved in specialized metabolism in *C. acuminata*. Tandem duplications of genes involved in specialized metabolism have been reported previously [39, 40] and, via divergence either in the coding region or promoter sequence that leads to neo- and sub-functionalization at the enzymatic or expression level, respectively, have been shown to contribute to the extensive chemical diversity within a species [40, 41].

The *C. acuminata* genome can also be used to facilitate our understanding of the mechanisms by which camptothecin production evolved independently in distinct taxa such as *C. acuminata* (Nyssaceae) and *O. pumila* (Rubiaceae). For example, a comparative analysis of *C. acuminata* and *O. pumila* may be highly informative in not only delineating genes involved in camptothecin biosynthesis but also in revealing key evolutionary events that led to biosynthesis of this critical natural product across a wide phylogenetic distance. As noted above, camptothecin is cytotoxic, and, as a consequence, derivatives of camptothecin are used as anti-cancer drugs. Perhaps most exciting, the ability to decipher the full camptothecin biosynthetic pathway will yield molecular reagents that can be used to not only synthesize camptothecin in heterologous systems such as yeast, but also produce less toxic analogs with novel pharmaceutical applications.

## Availability of supporting information

Raw genomic sequence reads and transcriptome reads derived from root tissues are available in the NCBI Sequence Read Archive under project number PRJNA361128. All other RNA-seq transcriptome reads were from Bioproject PRJNA80029 [4]. The genome assembly and annotation are available in the Dryad Digital Repository [42] and through the Medicinal Plant Genomics Resource [43] via a genome browser and search and analysis tools.

## Additional files

Table S1: RNA-sequencing libraries used in this study.

Table S2: Orthologous groups of genes from *Camptotheca acuminata* and 3 other plant species, available as a separate XLS file.

Table S3: P450 paralogous genes in *Camptotheca acuminata*, available as a separate XLS file.

Table S4: Expression abundance matrix (fragments per kbp exon model per million mapped reads) from different tissues of *Camptotheca acuminata*, available as a separate XLS file.

## Abbreviations

7DLH: 7-deoxyloganic acid 7-hydroxylase; CRL: custom repeat library; GOR: 8-hydroxygeraniol oxidoreductase; MEP: 2-*C*-methyl-D-erythritol 4-phosphate/1-deoxy-D-xylulose 5-ph-osphate; NCBI: National Center for Biotechnology Information; NMT: 16-hydroxy-2,3-dihydro-3-hydroxytabersonine N-methyltransferase; RNA-seq: RNA-sequencing.

## Competing interests

The authors have declared that no competing interests exist.

## Funding

Funding for this work was provided in part by a grant to C.R.B. and D.D.P. from the National Institute of General Medical Sciences (1RC2GM092521) and funds to C.R.B. and D.D.P. from Michigan State University. The funders had no role in study design, data collection and analysis, decision to publish, or preparation of the manuscript.

## Author contributions

C.R.B. oversaw the project. D.Z. performed the genome assembly, assisted in genome annotation, and analyzed data. J.H. annotated the genome and analyzed data. E.C., G.P., and K.W.R. constructed libraries and analyzed data. B.V. analyzed data. D.D.P. provided intellectual oversight. D.Z., J.H., and C.R.B. wrote the manuscript.

## Supplementary Material

GIGA-D-17-00067_Original-Submission.pdf

GIGA-D-17-00067_Revision-1.pdf

GIGA-D-17-00067_Revision-2.pdf

Response-to-Reviewer-Comments_Original-Submission.pdf

Response-to-Reviewer-Comments_Revision-1.pdf

Reviewer-1-Report-(Original-Submission).pdf

Reviewer-1-Report-(Revision-1).pdf

Reviewer-2-Report-(Original-Submission).pdf

Additional file Table S1: RNA-sequencing libraries used in this study.

Additional file Table S2: Orthologous groups of genes from *Camptotheca acuminata* and 3 other plant species, available as a separate XLS file.

Additional file Table S3: P450 paralogous genes in *Camptotheca acuminata*, available as a separate XLS file.

Additional file Table S4: Expression abundance matrix (fragments per kbp exon model per million mapped reads) from different tissues of *Camptotheca acuminata*, available as a separate XLS file.
