# Supplementary material for: De novo genome assembly of Camptotheca acuminata, a natural source of the anti-cancer compound camptothecin
Source: Gigascience. 2017 Jul 24;6(9):1–7. doi: 10.1093/gigascience/gix065 (PMC5737489; doi:10.1093/gigascience/gix065)
Supplement: GIGA-D-17-00067_Revision-1.pdf [file gix065_giga-d-17-00067_revision-1.pdf]

Manuscript Click here to download Manuscript Camptotheca_Ms_v16.docx

De novo genome assembly of *Camptotheca acuminata*, a natural source of the anti-cancer compound camptothecin

Dongyan Zhao[1], John P. Hamilton[1], Gina M. Pham[1], Emily Crisovan[1], Krystle Wiegert-Rininger[1], Brieanne Vaillancourt[1], Dean DellaPenna[2], and C. Robin Buell[1*]

[1]Department of Plant Biology, Michigan State University, East Lansing, MI 48824 USA

[2]Department of Biochemistry & Molecular Biology, Michigan State University, East Lansing, MI 48824 USA

Email addresses: Dongyan Zhao <zhaodon4@msu.edu>, John P. Hamilton <jham@msu.edu>, Gina M. Pham <phamgina@msu.edu>, Emily Crisovan <pankeyem@msu.edu>, Krystle Wiegert-Rininger <wiegertk@msu.edu>, Brieanne Vaillancourt <vaillan6@msu.edu>, Dean Dellapenna <dellapen@msu.edu>, C Robin Buell <buell@msu.edu>

*Correspondence should be addressed to: C. Robin Buell, buell@msu.edu

Manuscript type: Data note

Note: Reviewers can access the genome sequence and annotation using the following temporary URL: http://datadryad.org/review?doi=doi:10.5061/dryad.nc8qr.

Abstract

Background: *Camptotheca acuminata* is one of a limited number of species that produce camptothecin, a pentacyclic quinoline alkaloid with anti-cancer activity due to its ability to inhibit DNA topoisomerase. While transcriptome studies have been performed previously with various camptothecin-producing species, no genome sequence for a camptothecin-producing species is available to date.

Findings: We generated a high quality *de novo* genome assembly for *C. acuminata* representing 403,174,860 bp on 1,394 scaffolds with an N50 scaffold size of 1,752 kbp. Quality assessments of the assembly revealed robust representation of the genome sequence including genic regions. Using a novel genome annotation method, we annotated 31,825 genes encoding 40,332 gene models. Based on sequence identity and orthology with validated genes from *Catharanthus roseus* as well as Pfam searches, we identified candidate orthologs for genes potentially involved in camptothecin biosynthesis. Extensive gene duplication including tandem duplication was widespread in the *C. acuminata* genome with 3,315 genes belonging to 1,245 tandem duplicated gene clusters.

Conclusions: To our knowledge, this is the first genome sequence for a camptothecin-producing species, and access to the *C. acuminata* genome will permit not only discovery of genes encoding the camptothecin biosynthetic pathway but also reagents that can be used for heterologous expression of camptothecin and camptothecin analogs with novel pharmaceutical applications.

Keywords: *Camptotheca acuminata*, camptothecin, genome assembly, genome annotation, tandem duplications

Data Description

Background information on camptothecin, a key anti-cancer natural product

Camptotheca acuminata Decne, also known as the Chinese Happy Tree (Figure 1), is an eudicot asterid Cornales tropical tree species within the Nyssaceae family [1] that also contains *Nyssa* spp (tupelo) and *Davidia involucrate* (dove tree); no genome sequence is available for any member of this family. *C. acuminata* is one of a limited number of plant species that produce camptothecin, a pentacyclic quinoline alkaloid (Figure 2A) with anti-cancer activity due to its ability to inhibit DNA topoisomerase [2]. Due to poor solubility, derivatives such as irinotecan and topotecan, rather than camptothecin are currently in use as approved cancer drugs. The significance of these derivatives as therapeutics is highlighted by the listing of irinotecan on the World Health Organization Model List of Essential Medicines [3]. While transcriptome studies have been performed previously with various camptothecin-producing species including *C. acuminata* and *Ophiorrhiza pumila* (e.g., [4-6]), no genome sequence for a camptothecin-producing species is available to date. We report on the assembly and annotation of the *C. acuminata* genome, the characterization of genes implicated in camptothecin biosynthesis, and highlight the extent of gene duplication that provides new templates for gene diversification.

RNA isolation, library construction, sequencing, and transcriptome assembly

Transcriptome assemblies were constructed using nine developmental RNA-sequencing (RNA-seq) datasets described in a previous study [4] that included immature bark, cotyledons, immature flower, immature fruit, mature fruit, mature leaf, root, upper stem, and lower stem. Adapters and low-quality nucleotides were removed from the RNA-seq reads using Cutadapt (v1.8) [7] and contaminating ribosomal RNA reads were removed. Cleaned reads from all nine libraries were assembled using Trinity (v20140717) [8] with a normalization factor of 50x using default parameters. Contaminant transcripts (5,669 total) were identified by searching the *de novo* transcriptome assembly against the National Center for Biotechnology Information (NCBI) non-redundant nucleotide database using BLAST+ (v2.2.30) [9, 10] with an E-value cutoff of 1e-5; transcripts with their best hits being a non-plant sequence were removed from the transcriptome.

For additional transcript support for use in a genome-guided transcriptome assembly to support genome annotation, strand-specific RNA-seq reads were generated by isolating RNA from root tissues and sequencing of Kappa TruSeq Stranded libraries on an Illumina HiSeq 2500 platform generating 150 nt paired-end reads (BioSample ID: SAMN06229771). Root RNA-seq reads were assessed for quality using FASTQC (v0.11.2) [11] using default parameters and cleaned as described above.

DNA isolation, library construction, and sequencing

The genome size of *C. acuminata* was estimated at 516 Mb using flow cytometry, suitable for *de novo* assembly using the Illumina platform. DNA was extracted from young leaves of *C. acuminata* at the vegetative growth stage using CTAB [12]. Multiple Illumina-compatible paired-

end libraries (Table 1) with insert sizes ranging from 180-609 bp were constructed as described previously [13] and sequenced to 150 nt in paired-end mode on an Illumina HiSeq2000. Mate-pair libraries (Table 1) with size ranges of 1.3-8.9 kb were made using the Nextera Kit (Illumina, San Diego CA) as per manufacturer's instructions and sequenced to 150 nt in paired-end mode on an Illumina HiSeq2000.

Genome assembly

Paired-end reads (Table 1) were assessed for quality using FASTQC (v0.11.2) [11] using default parameters, cleaned for adapters and low quality sequences using Cutadapt (v1.8) [7] and only reads in pairs with each read ≥25 nt were retained for genome assembly. Mate pair libraries (Table 1) were processed using NextClip (v1.3.1) [14] and only reads from Categories A, B, C were used for the assembly. Using ALLPATHS-LG (v44837) [15] with default parameters, two paired-end read libraries (180 and 268 bp insert libraries) and all five mate pair libraries (Table 1) were used to generate an initial assembly of 403.2 Mb with an N50 contig size of 108 kbp and an N50 scaffold size of 1,752 kbp (Tables 1 and 2). Gaps (5,076) in this initial assembly were filled using SOAP GapCloser (v1.12r6) [16] with four independent paired-end libraries (352, 429, 585, and 609 bp inserts, Table 1); 12,468,362 bp of the estimated 16,471,841 bp of gaps was filled leaving a total of 3,825 gaps (3,772,191 Ns). The assembly was checked for contaminant sequences based on alignments to the NCBI non-redundant nucleotide database using BLASTN (E-value = 1e-5) [10]; a single scaffold of 5,156 bp that matched a bacterium sequence with 100% coverage and 100% identity was removed. Subsequently, five scaffolds less than 1 kbp

were removed resulting in the final assembly of 403,174,860 bp comprised of 1,394 scaffolds with an N50 scaffold size of 1,752 kbp (Tables 1 and 2) and 0.9% Ns.

Quality assessments revealed a robust high quality assembly with 98% of the paired-end genomic sequencing reads aligning to the assembly, of which, 99.97% aligned concordantly. With respect to genic representation, 95.3% of RNA-seq-derived transcript assemblies [4] and 74,119 of 74,682 (99%) pyrosequencing transcript reads from a separate study [5] aligned to the genome assembly. A total of 93.6% of conserved Embryophyta BUSCO proteins were present in the assembly as full-length sequences with an additional 2.5% of the Embryophyta proteins fragmented [17].

Genome annotation

We used a novel genome annotation method to generate high quality annotation of the *C. acuminata* genome in which we repeat masked the genome, trained an *ab initio* gene finder with a genome-guided transcript assembly, and then refined the gene models using additional genome-guided transcript assembly evidence to generate a high quality gene model set. We first created a *C. acuminata* specific custom repeat library (CRL) using MITE-Hunter (v2011) [18] and RepeatModeler (v1.0.8) [19]. Protein coding genes were removed from each repeat library using ProtExcluder.pl (v1.1) [20] and combined into a single CRL, which hard-masked 143.6 Mb (35.6%) of the assembly as repetitive sequence using RepeatMasker (v4.0.6) [21]. Cleaned root RNA-seq reads (Table S1, BioSample ID: SAMN06229771) were aligned to the genome assembly using TopHat2 (v2.0.13) [22] in strand-specific mode with a minimum intron length of 20 bp and a maximum intron length of 20 kb; the alignments were then used to create a genome-guided

transcriptome assembly using Trinity (v2.2.0) [23]. The RNA-seq alignments were used to train AUGUSTUS (v3.1) [24] and gene predictions were generated with AUGUSTUS [25] using the hard-masked assembly. Gene model structures were refined by incorporating evidence from the genome-guided transcriptome assembly using PASA2 (v2.0.2) [26, 27]; with the parameters: MIN_PERCENT_ALIGNED=90, MIN_AVG_PER_ID=99. After annotation comparison, models that PASA identified as being merged and a subset of candidate camptothecin biosynthetic pathways genes identified as mis-annotated were manually curated. The final high-confidence gene model set consists of 31,825 genes encoding 40,332 gene models. Functional annotation was assigned using a custom pipeline using WU-BLASTP [28] searches against the *Arabidopsis thaliana* annotation (TAIR10; [29]) and Swiss-Prot plant proteins (downloaded on 08-17-2015), and a search against Pfam (v29) using HMMER (v3.1b2) [30]. This resulted in 34,143 gene models assigned a putative function, 2,011 annotated as conserved hypothetical, and 4,178 annotated as hypothetical.

C. acuminata is insensitive to camptothecin due to mutations within its own DNA topoisomerase [31] and we identified two topoisomerase genes in our annotated gene set, one of which matches the published *C. acuminata* topoisomerase (99.78% identity, 100% coverage) and includes the two mutations that confer resistance to camptothecin (Figure 2B), one mutation is specific in *C. acuminata* and the other is present in both *C. acuminata* and two camptothecin-producing *Ophiorrhiza* species. Further quality assessments of our annotation with 35 nuclear-encoded *C. acuminata* genes available from GenBank revealed an average identity of 99.5% with 100% coverage in our annotated proteome while a single gene encoding 1-deoxy-D-xylulose 5-phosphate reductoisomerase (ABC86579.1) had 88.2% identity with 100%

coverage that may be attributable to differences in genotypes. One mRNA reported to encode a putative strictosidine beta-D-glucosidase (AES93119.1) was found to have a retained intron that when removed, aligned with 99.3% identity yet reduced coverage (66%) as it was located at the end of a short scaffold. Collectively, the concordant alignment of whole genome shotgun sequence reads to the assembly, the high representation of genic regions as assessed by independent transcriptome datasets (RNA-seq and pyrosequencing) as well as the core Embryophyta BUSCO proteins, when coupled with the high quality gene models as revealed through alignments with cloned *C. acuminata* genes indicate that we have not only generated a high quality genome assembly for *C. acuminata* but also a robust set of annotated gene models.

Gene duplication and orthology analyses

During our annotation efforts, it was readily apparent that there was substantial gene duplication including tandem gene duplication in the *C. acuminata* genome. Paralogous clustering of the *C. acuminata* proteome revealed 5,768 paralogous groups containing 17,957 genes. We identified tandem gene duplications in the *C. acuminata* genome based on if: 1) two or more *C. acuminata* genes were present within an orthologous/paralogous group; 2) there were no more than 10 genes in between on a single scaffold; and 3) the pairwise gene distance was less than 100 kbp [32]. Under these criteria, a total of 3,315 genes belonging to 1,245 tandem duplicated gene clusters were identified. Gene ontology analysis showed that tandem duplicated genes are significantly enriched in “response to stress” ($p < 0.0001$, χ^2 test) while under-represented in most other processes, especially “other cellular processes” and “cell organization and biogenesis” ($p < 0.0001$, χ^2 test).

To our knowledge, *C. acuminata* is the first species within the Nyssaceae family with a genome sequence. To better understand the evolutionary relationship of *C. acuminata* with other asterids and angiosperms, we identified orthologous and paralogous groups using our annotated *C. acuminata* proteome and the proteomes of five other key species (*Arabidopsis thaliana*, *Amborella trichopoda*, *Vitis vinifera*, *Oryza sativa*, and *Catharanthus roseus*) using OrthoFinder (v0.7.1) [33] with default parameters. A total of 12,459 orthologous groups containing at least a single *C. acuminata* protein were identified with 8,521 orthologous groups common to all six species (Figure 3; Table S2). Interestingly, *C. acuminata* contains the least number of singleton genes (7,177) among the six species, and gene ontology analysis demonstrated that these genes were highly enriched in “transport”, “response to stress”, and “other cellular and biological processes” ($p < 0.0001$, χ^2 test) while dramatically under-represented in “unknown biological processes” ($p < 0.0001$, χ^2 test), suggesting these genes may be involved in stress responses and other processes specific to *C. acuminata*.

Uses for the *C. acuminata* genome sequence and annotation

Generation of a high-quality genome sequence and annotation dataset for *C. acuminata* will facilitate discovery of genes encoding camptothecin biosynthesis as physical clustering can be combined with sequence similarity and co-expression data to identify candidate genes, an approach that has been extremely useful in identifying genes in specialized metabolism in a number of plant species (see [34-36]). In *C. acuminata*, geranylgeranyl diphosphate from the 2-*C*-methyl-D-erythritol 4-phosphate/1-deoxy-D-xylulose 5-phosphate (MEP) pathway is used to generate secologanic acid via the iridoid pathway and tryptamine from tryptophan

decarboxylase are condensed by strictosidinic acid synthase to generate strictosidinic acid that is then converted into camptothecin in the alkaloid pathway via a set of unknown steps [37] (Figure 4A). *Catharanthus roseus*, Madagascar periwinkle, produces vinblastine and vincristine via the MEP and iridoid pathways for which all genes leading to the biosynthesis of the iridoid secologanin have been characterized [35]. Using sequence identity and coverage with characterized *C. roseus* genes from the MEP and iridoid pathway (Figure 4A), we were able to identify candidate genes for all steps in the MEP and iridoid pathway in *C. acuminata* (Table 3). The downstream steps in camptothecin biosynthesis subsequent to formation of strictosidinic acid involve a broad set of enzymes responsible for reduction and oxidation [37] and a total of 343 cytochrome P450s (60 paralogous gene clusters and 86 singletons; Table S3) were identified which can serve as candidates for the later steps in camptothecin biosynthesis.

Though not absolute, physical clustering of genes involved in specialized metabolism has been observed in a number of species across a number of classes of specialized metabolites [34, 38]. With an N50 scaffold size of 1,752 kbp, we observed several instances of physical clustering of genes with homology to genes involved in monoterpene indole alkaloid biosynthesis which may produce related compounds in *C. acuminata*. Using characterized genes involved in the biosynthesis of vinblastine and vincristine from *C. roseus* as queries [35] (Figure 4A, Table 3), we identified a single *C. acuminata* scaffold (907 kbp, 86 genes; Figure 4B) that encoded genes with sequence identity to isopentenyl diphosphate isomerase II within the MEP pathway, 8-hydroxygeraniol oxidoreductase (GOR, three complete and one partial paralogs), 7-deoxyloganic acid 7-hydroxylase (7DLH) within the iridoid pathway, and a protein with homology to *C. roseus* 16-hydroxy-2,3-dihydro-3-hydroxytabersonine N-methyltransferase

(NMT) within the alkaloid pathway suggesting that access to a high contiguity genome assembly may facilitate discovery of genes involved in specialized metabolism in *C. acuminata*. Tandem duplications of genes involved in specialized metabolism have been reported previously [39, 40] and via divergence either in the coding region or promoter sequence which lead to neo- and sub-functionalization at the enzymatic or expression level, respectively, have been shown to contribute to the extensive chemical diversity within a species [40, 41].

The *C. acuminata* genome can also be used to facilitate our understanding of the mechanisms by which camptothecin production evolved independently in distinct taxa such as *C. acuminata* (Nyssaceae) and *O. pumila* (Rubiaceae). For example, a comparative analysis of *C. acuminata* and *O. pumila* may be highly informative in not only delineating genes involved in camptothecin biosynthesis but also in revealing key evolutionary events that led to biosynthesis of this critical natural product across a wide phylogenetic distance. As noted above, camptothecin is cytotoxic and as a consequence, derivatives of camptothecin are used as anti-cancer drugs. Perhaps most exciting, the ability to decipher the full camptothecin biosynthetic pathway will yield molecular reagents that can be used to not only synthesize camptothecin in heterologous systems such as yeast, but also produce less toxic analogs with novel pharmaceutical applications.

Availability of Supporting Information

Raw genomic sequence reads and transcriptome reads derived from root tissues are available in the NCBI Sequence Read Archive under project number PRJNA361128. All other RNA-seq transcriptome reads were from Bioproject PRJNA80029 [4]. The genome assembly and

annotation are available in the Dryad Digital Repository under doi (to be released upon publication), through the Medicinal Plant Genomics Resource [42] via a genome browser and search and analysis tools, and GigaDB.

Note: Reviewers can access the genome sequence and annotation using the following temporary URL: http://datadryad.org/review?doi=doi:10.5061/dryad.nc8qr.

Abbreviations

2-*C*-methyl-D-erythritol 4-phosphate/1-deoxy-D-xylulose 5-phosphate (MEP), 7-deoxyloganic acid 7-hydroxylase (7DLH), 8-hydroxygeraniol oxidoreductase (GOR), 16-hydroxy-2,3-dihydro-3-hydroxytabersonine N-methyltransferase (NMT), custom repeat library (CRL), National Center for Biotechnology Information (NCBI), RNA-sequencing (RNA-seq)

Competing Interests

The authors have declared that no competing interests exists.

Author Contributions

CRB oversaw the project. DZ performed the genome assembly, assisted in genome annotation and analyzed data. JH annotated the genome and analyzed data. EC, GP, and KWR constructed libraries and analyzed data. BV analyzed data. DDP provided intellectual oversight. DZ, JH, and CRB wrote the manuscript.

Acknowledgements

Funding for this work was provided in part by a grant to CRB and DDP from the National Institute of General Medical Sciences (1RC2GM092521) and funds to CRB and DDP from Michigan State University. The funders had no role in study design, data collection and analysis, decision to publish, or preparation of the manuscript.

Figure Legends

Figure 1. *Camptotheca acuminata* Decne, the Chinese Happy Tree, is a member in the Nyssaceae family that produces the anticancer compound camptothecin.

Figure 2. Genome aspects of *Camptotheca acuminata*. (A) Structure of camptothecin. (B) Key amino acid mutations (red rectangles) in DNA topoisomerase I in camptothecin-producing and non-producing species and their phylogenetic relationship.

Figure 3. Venn diagram showing orthologous and paralogous groups between *Camptotheca acuminata*, *Amborella trichopoda*, *Oryza sativa*, *Arabidopsis thaliana*, *Vitis vinifera*, and *Catharanthus roseus.*

Figure 4. Key portions of the proposed camptothecin biosynthetic pathway and an example of physical clustering of candidate genes in *Camptotheca acuminata*. (A) The methylerythritol phosphate (MEP) pathway (green), iridoid pathway (blue), and condensation of secologanic acid with tryptamine via strictosidinic acid synthase (STRAS) to form strictosidinic acid prior to downstream dehydration, reduction, and oxidation steps yielding camptothecin. DXS, 1-deoxy-D-xylulose 5-phosphate synthase 2; DXR, 1-deoxy-D-xylulose-5-phosphate reductoisomerase; CMS, 4-diphosphocytidyl-methylerythritol 2-phosphate synthase; CMK, 4-diphosphocytidyl-2-C-methyl-D-erythritol kinase; MCS, 2C-methyl-D-erythritol 2,4-cyclodiphosphate synthase; HDS, GCPE protein; HDR, 1-hydroxy-2-methyl-butenyl 4-diphosphate reductase; IPI, plastid isopentenyl pyrophosphate, dimethylallyl pyrophosphate isomerase; GPPS, geranyl pyrophosphate synthase; GES, plastid geraniol synthase; G8H, geraniol 8-hydroxylase; GOR, 8-hydroxygeraniol oxidoreductase; CYC1, iridoid cyclase 1; 7-DLS,

7-deoxyloganetic acid synthase; 7-DLGT, 7-deoxyloganetic acid glucosyltransferase; 7-DLH, 7-deoxyloganic acid hydroxylase; SLAS, secologanic acid synthase; TDC, tryptophan decarboxylase. **(B) Physical clustering of homologs of genes involved in the methylerythritol phosphate, iridoid, and alkaloid biosynthetic pathways of *Catharanthus roseus* on scaffold 151 of *C. acuminata*.** GOR: 8-hydroxygeraniol oxidoreductase; NMT: 16-hydroxy-2,3-dihydro-3-hydroxytabersonine N-methyltransferase; 7DLH: 7-deoxyloganic acid 7-hydroxylase; IPP2: isopentenyl diphosphate isomerase II. Gene IDs are below the arrows.

Table 1. Input libraries and sequences for *de novo* assembly of the *Camptotheca acuminata* genome.

BioProject ID	BioSample ID	Fragment size (bp)	No. of cleaned read pairs	Use
**Paired end**				
PRJNA361128	SAMN06220985	180	96,955,546	ALLPATHS-LG assembly
PRJNA361128	SAMN06220986	268	89,381,055	ALLPATHS-LG assembly
PRJNA361128	SAMN06220987	352	61,207,691	GapCloser
PRJNA361128	SAMN06220988	429	50,688,562	GapCloser
PRJNA361128	SAMN06220989	585	21,856,610	GapCloser
PRJNA361128	SAMN06220990	609	22,217,954	GapCloser
**Mate pair**				
PRJNA361128	SAMN06220991	8,111	9,923,643	ALLPATHS-LG assembly
PRJNA361128	SAMN06220992	7,911	7,652,519	ALLPATHS-LG assembly
PRJNA361128	SAMN06220993	1,377	12,800,554	ALLPATHS-LG assembly
PRJNA361128	SAMN06220994	3,179	13,138,503	ALLPATHS-LG assembly
PRJNA361128	SAMN06220995	8,879	13,599,241	ALLPATHS-LG assembly

All libraries were sequenced in paired end mode generating 150 nt reads.

Table 2. Metrics of the final assembly of *Camptotheca acuminata* genome.

Metric	Value
Total scaffold length (bp)	403,174,860
Total no. of scaffolds (bp)	1,394
Maximum scaffold length (bp)	8,423,530
Minimum scaffold length (bp)	1,002
N50 scaffold size (bp)	1,751,747
N50 contig size (bp)	107,594
No. Ns	3,772,191 (0.9%)
No. gaps	3,825

Table 3. Identification of candidate camptothecin biosynthetic pathway genes in the *Camptotheca acuminata* genome as revealed by sequence identity and coverage with characterized genes from the 2-*C*-methyl-D-erythritol 4-phosphate/1-deoxy-D-xylulose 5-phosphate and iridoid biosynthetic pathways from *Catharanthus roseus*.

Description	Abbreviation	Protein	Camptotheca Gene ID	% coverage	% identity
**MEP**					
1-deoxy-D-xylulose 5-phosphate synthase 2	DXS	ABI35993.1	Cac_g024944.t1	98	77.60
1-deoxy-D-xylulose-5-phosphate reductoisomerase	DXR	AAF65154.1	Cac_g016318.t1	100	88.82
4-diphosphocytidyl-methylerythritol 2-phosphate synthase	CMS	ACI16377.1	Cac_g018722.t1	88	77.82
4-diphosphocytidyl-2-C-methyl-D-erythritol kinase	CMK	ABI35992.1	Cac_g021688.t1	99	76.17
2C-methyl-D-erythritol 2,4-cyclodiphosphate synthase	MCS	AAF65155.1	Cac_g008169.t1	100	73.77
GCPE protein	HDS	AAO24774.1	Cac_g022763.t1	100	88.65
1-hydroxy-2-methyl-butenyl 4-diphosphate reductase	HDR	ABI30631.1	Cac_g014659.t1	100	83.77
plastid isopentenyl pyrophosphate:dimethylallyl pyrophosphate isomerase	IPI	ABW98669.1	Cac_g008847.t1	76	91.06
geranyl pyrophosphate synthase	GPPS	ACC77966.1	Cac_g026508.t1	51	76.50
**Iridoid**					
geraniol 8-hydroxylase	G8H	CAC80883.1	Cac_g017987.t1	95	76.71
8-hydroxygeraniol oxidoreductase	GOR	AHK60836.1	Cac_g027560.t1	100	71.69
iridoid synthase	ISY	AFW98981.1	Cac_g006027.t1	100	65.65
iridoid oxidase	IO	AHK60833.1	Cac_g032709.t1	97	78.44
UDP-glucose iridoid glucosyltransferase	7DLGT	BAO01109.1	Cac_g008744.t1	100	77.11
7-deoxyloganic acid 7-hydroxylase	7DLH	AGX93062.1	Cac_g012663.t1	96	69.58
loganic acid methyltransferase	LAMT	ABW38009.1	Cac_g005179.t1	95	53.91
secologanin synthase	SLS	AAA33106.1	Cac_g012666.t1	99	64.94

Note: Only the top hit from the BLAST search is presented.

References

1. Angiosperm Phylogeny Group III. An update of the Angiosperm Phylogeny Group classification for the orders and families of flowering plants: APG III. Botanical Journal of the Linnean Society. 2009;161:105-21.
2. Lorence A, Nessler, C.L. Molecules of Interest: Camptothecin, over four decades of surprising findings. Phytochemistry. 2004; 65:2735–49.
3. World Health Organization: 19th WHO Model List of Essential Medicines. http://www.who.int/medicines/publications/essentialmedicines/EML2015_8-May-15.pdf. Accessed 26 March 2017.
4. Gongora-Castillo E, Childs KL, Fedewa G, Hamilton JP, Liscombe DK, Magallanes-Lundback M, et al. Development of transcriptomic resources for interrogating the biosynthesis of monoterpene indole alkaloids in medicinal plant species. PLoS One. 2012;7 12:e52506. doi:10.1371/journal.pone.0052506.
5. Sun Y, Luo H, Li Y, Sun C, Song J, Niu Y, et al. Pyrosequencing of the *Camptotheca acuminata* transcriptome reveals putative genes involved in camptothecin biosynthesis and transport. BMC Genomics. 2011;12:533. doi:10.1186/1471-2164-12-533.
6. Yamazaki M, Mochida K, Asano T, Nakabayashi R, Chiba M, Udomson N, et al. Coupling deep transcriptome analysis with untargeted metabolic profiling in *Ophiorrhiza pumila* to further the understanding of the biosynthesis of the anti-cancer alkaloid camptothecin and anthraquinones. Plant Cell Physiol. 2013;54 5:686-96. doi:10.1093/pcp/pct040.
7. Martin M. Cutadapt removes adapter sequences from high-throughput sequencing reads. EMBnetjournal. 2011;17 1 doi:http://dx.doi.org/10.14806/ej.17.1.200.
8. Haas BJ, Papanicolaou A, Yassour M, Grabherr M, Blood PD, Bowden J, et al. De novo transcript sequence reconstruction from RNA-seq using the Trinity platform for reference generation and analysis. Nat Protoc. 2013;8 8:1494-512. doi:10.1038/nprot.2013.084.
9. Altschul SF, Madden TL, Schaffer AA, Zhang J, Zhang Z, Miller W, et al. Gapped BLAST and PSI-BLAST: a new generation of protein database search programs. Nucleic Acids Res. 1997;25 17:3389-402.
10. Camacho C, Coulouris G, Avagyan V, Ma N, Papadopoulos J, Bealer K, et al. BLAST+: architecture and applications. BMC Bioinformatics. 2009;10:421. doi:10.1186/1471-2105-10-421.
11. FastQC. http://www.bioinformatics.babraham.ac.uk/projects/fastqc/. Accessed 26 March 2017.
12. Saghai-Maroof MA, Soliman KM, Jorgensen RA and Allard RW. Ribosomal DNA spacer-length polymorphisms in barley - Mendelian inheritance, chromosomal location, and population-dynamics. PRoc Natl Acad USA. 1984;81 24:8014-8. doi:Doi 10.1073/Pnas.81.24.8014.
13. Hardigan MA, Crisovan E, Hamilton JP, Kim J, Laimbeer P, Leisner CP, et al. Genome Reduction Uncovers a Large Dispensable Genome and Adaptive Role for Copy Number Variation in Asexually Propagated *Solanum tuberosum*. Plant Cell. 2016;28 2:388-405. doi:10.1105/tpc.15.00538.
14. Leggett RM, Clavijo BJ, Clissold L, Clark MD and Caccamo M. NextClip: an analysis and read preparation tool for Nextera Long Mate Pair libraries. Bioinformatics. 2014;30 4:566-8. doi:10.1093/bioinformatics/btt702.
15. Gnerre S, Maccallum I, Przybylski D, Ribeiro FJ, Burton JN, Walker BJ, et al. High-quality draft assemblies of mammalian genomes from massively parallel sequence data. Proc Natl Acad Sci U S A. 2011;108 4:1513-8. doi:1017351108 [pii]10.1073/pnas.1017351108.

16. Luo R, Liu B, Xie Y, Li Z, Huang W, Yuan J, et al. SOAPdenovo2: an empirically improved memory-efficient short-read de novo assembler. Gigascience. 2012;1 1:18. doi:10.1186/2047-217X-1-18.
17. Simao FA, Waterhouse RM, Ioannidis P, Kriventseva EV and Zdobnov EM. BUSCO: assessing genome assembly and annotation completeness with single-copy orthologs. Bioinformatics. 2015;31 19:3210-2. doi:10.1093/bioinformatics/btv351.
18. Han Y and Wessler SR. MITE-Hunter: a program for discovering miniature inverted-repeat transposable elements from genomic sequences. Nucleic Acids Res. 2010;38 22:e199. doi:10.1093/nar/gkq862.
19. Repeat Modeler. http://www.repeatmasker.org/. Accessed 26 March 2017.
20. Campbell MS, Law M, Holt C, Stein JC, Moghe GD, Hufnagel DE, et al. MAKER-P: a tool kit for the rapid creation, management, and quality control of plant genome annotations. Plant Physiol. 2014;164 2:513-24. doi:10.1104/pp.113.230144.
21. RepeatMasker. http://www.repeatmasker.org/. Accessed 26 March 2017.
22. Kim D, Pertea G, Trapnell C, Pimentel H, Kelley R and Salzberg SL. TopHat2: accurate alignment of transcriptomes in the presence of insertions, deletions and gene fusions. Genome Biol. 2013;14 4:R36. doi:10.1186/gb-2013-14-4-r36.
23. Grabherr MG, Haas BJ, Yassour M, Levin JZ, Thompson DA, Amit I, et al. Full-length transcriptome assembly from RNA-Seq data without a reference genome. Nature Biotechnology. 2011;29 7:644-52. doi:10.1038/nbt.1883.
24. Stanke M, Schoffmann O, Morgenstern B and Waack S. Gene prediction in eukaryotes with a generalized hidden Markov model that uses hints from external sources. BMC Bioinformatics. 2006;7:62.
25. Stanke M and Morgenstern B. AUGUSTUS: a web server for gene prediction in eukaryotes that allows user-defined constraints. Nucleic Acids Res. 2005;33 Web Server issue:W465-7. doi:10.1093/nar/gki458.
26. PASA2. http://pasapipeline.github.io/. Accessed 26 March 2017.
27. Haas BJ, Delcher AL, Mount SM, Wortman JR, Smith RK, Jr., Hannick LI, et al. Improving the Arabidopsis genome annotation using maximal transcript alignment assemblies. Nucleic Acids Res. 2003;31 19:5654-66.
28. Altschul SF and Gish W. Local alignment statistics. Methods Enzymol. 1996;266:460-80.
29. The Arabidopsis Information Resource. Arabidopsis.org. Accessed 26 March 2017.
30. Eddy SR. Accelerated Profile HMM Searches. PLoS Comput Biol. 2011;7 10:e1002195. doi:10.1371/journal.pcbi.1002195.
31. Sirikantaramas S, Yamazaki M and Saito K. Mutations in topoisomerase I as a self-resistance mechanism coevolved with the production of the anticancer alkaloid camptothecin in plants. Proc Natl Acad Sci U S A. 2008;105 18:6782-6. doi:0801038105 [pii]10.1073/pnas.0801038105.
32. Hanada K, Zou C, Lehti-Shiu MD, Shinozaki K and Shiu SH. Importance of lineage-specific expansion of plant tandem duplicates in the adaptive response to environmental stimuli. Plant Physiol. 2008;148 2:993-1003.
33. Emms DM and Kelly S. OrthoFinder: solving fundamental biases in whole genome comparisons dramatically improves orthogroup inference accuracy. Genome Biol. 2015;16:157. doi:10.1186/s13059-015-0721-2.
34. Nutzmann HW and Osbourn A. Gene clustering in plant specialized metabolism. Curr Opin Biotechnol. 2014;26:91-9. doi:10.1016/j.copbio.2013.10.009.
35. Kellner F, Kim J, Clavijo BJ, Hamilton JP, Childs KL, Vaillancourt B, et al. Genome-guided investigation of plant natural product biosynthesis. Plant J. 2015;82 4:680-92. doi:10.1111/tpj.12827.

36. Itkin M, Heinig U, Tzfadia O, Bhide AJ, Shinde B, Cardenas PD, et al. Biosynthesis of antinutritional alkaloids in solanaceous crops is mediated by clustered genes. Science. 2013;341 6142:175-9. doi:10.1126/science.1240230.
37. Sadre R, Magallanes-Lundback M, Pradhan S, Salim V, Mesberg A, Jones AD, et al. Metabolite Diversity in Alkaloid Biosynthesis: A Multilane (Diastereomer) Highway for Camptothecin Synthesis in Camptotheca acuminata. Plant Cell. 2016;28 8:1926-44. doi:10.1105/tpc.16.00193.
38. DellaPenna D and O'Connor SE. Plant science. Plant gene clusters and opiates. Science. 2012;336 6089:1648-9. doi:10.1126/science.1225473.
39. Chae L, Kim T, Nilo-Poyanco R and Rhee SY. Genomic signatures of specialized metabolism in plants. Science. 2014;344 6183:510-3. doi:10.1126/science.1252076.
40. Kliebenstein DJ. A role for gene duplication and natural variation of gene expression in the evolution of metabolism. PLoS One. 2008;3 3:e1838. doi:10.1371/journal.pone.0001838.
41. Kliebenstein DJ, Lambrix VM, Reichelt M, Gershenzon J and Mitchell-Olds T. Gene duplication in the diversification of secondary metabolism: tandem 2-oxoglutarate-dependent dioxygenases control glucosinolate biosynthesis in Arabidopsis. Plant Cell. 2001;13 3:681-93.
42. The Medicinal Plant Genomics Resource. http://medicinalplantgenomics.msu.edu/. Accessed 26 March 2017.

Additional files

Supplemental tables:

Table S1. RNA-sequencing libraries used in this study.

BioProject ID	BioSample ID	Tissue	No. cleaned reads	Estimated bases
PRJNA80029	SAMN00255206	mature leaf	90,862,580	5,451,754,800
PRJNA80029	SAMN00255207	immature bark	84,537,958	5,072,277,480
PRJNA80029	SAMN00255208	root	88,940,668	5,336,440,080
PRJNA80029	SAMN00255215	young flower	71,435,806	4,286,148,360
PRJNA80029	SAMN00255216	immature fruit	84,250,338	5,055,020,280
PRJNA80029	SAMN00255217	mature fruit	47,811,342	2,868,680,520
PRJNA80029	SAMN00255222	cotyledons	74,037,722	4,442,263,320
PRJNA80029	SAMN00255223	upper stem	76,105,786	4,566,347,160
PRJNA80029	SAMN00255224	lower stem	72,680,940	4,360,856,400
PRJNA361128	SAMN06229771	root	55,435,804	7,224,198,331
Total			771,909,254	49,309,244,481

Table S2. Orthologous groups of genes from *Camptotheca acuminata* and five other plant species.

This is available as a separate XLS file

Table S3. P450 paralogous genes in *Camptotheca acuminata*.

This is available as a separate XLS file

Table S4. Expression abundance matrix (fragments per kbp exon model per million mapped reads) from different tissues of *Camptotheca acuminata*.

This is available as a separate XLS file

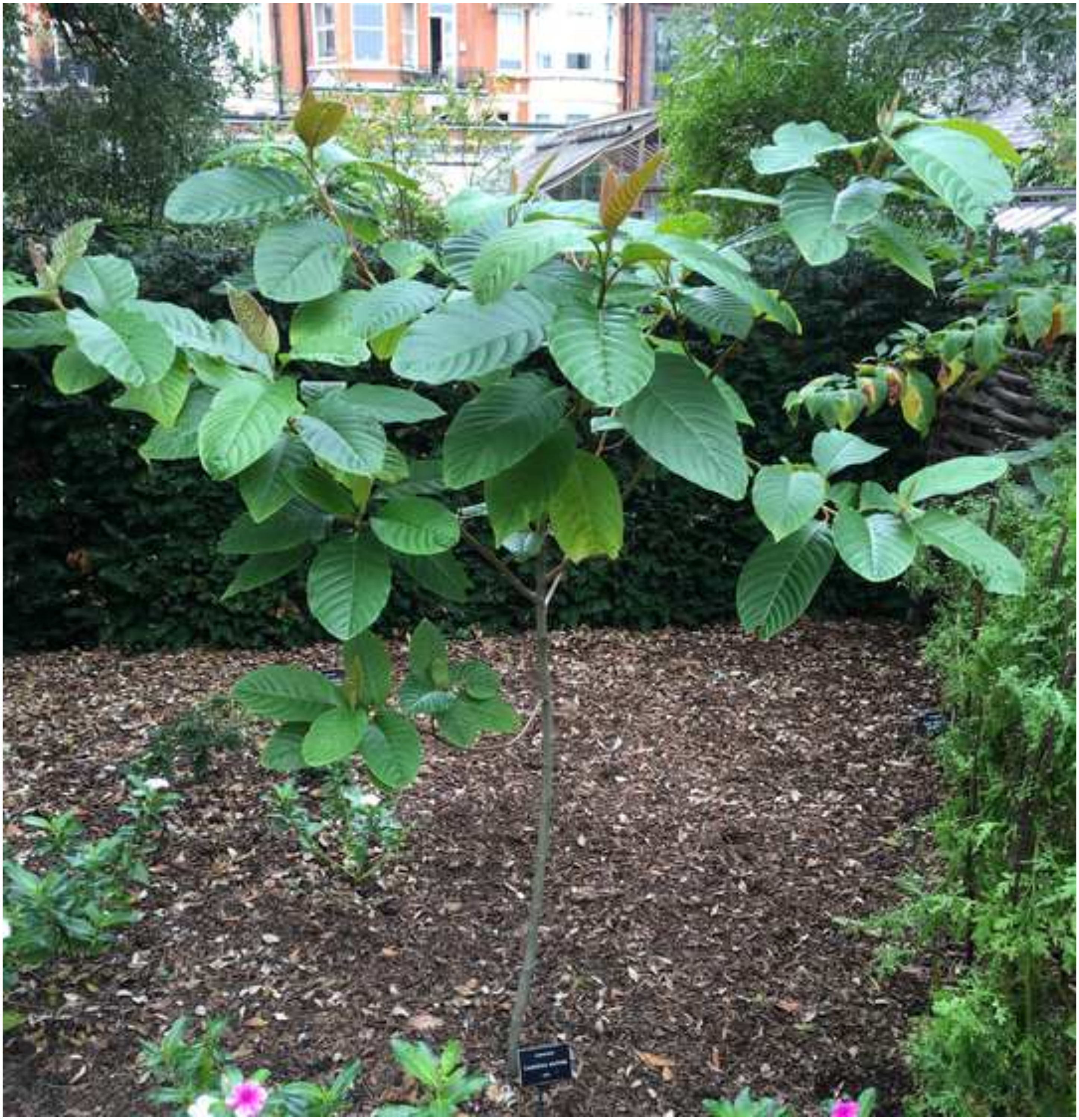

Figure 2

Click here to download Figure Fig_2.pptx

A

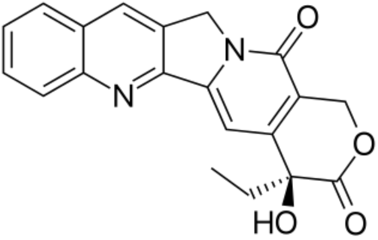

B

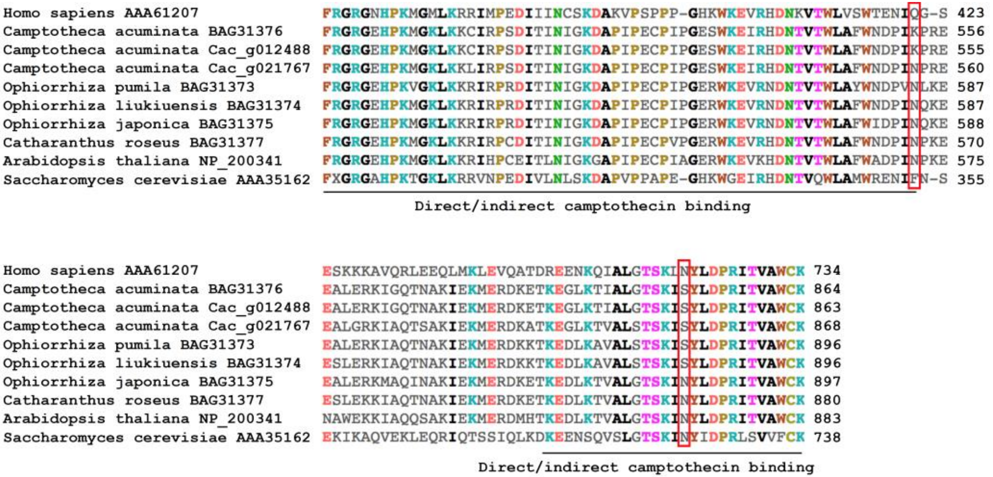

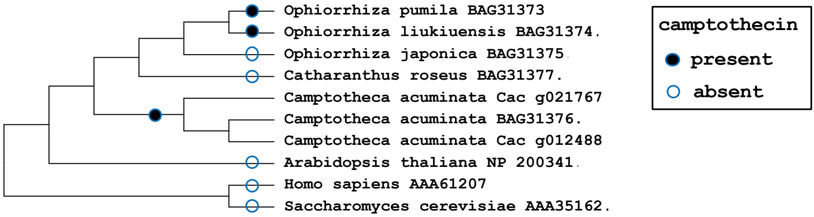

Figure 3

Click here to download Figure Fig_3.pptx

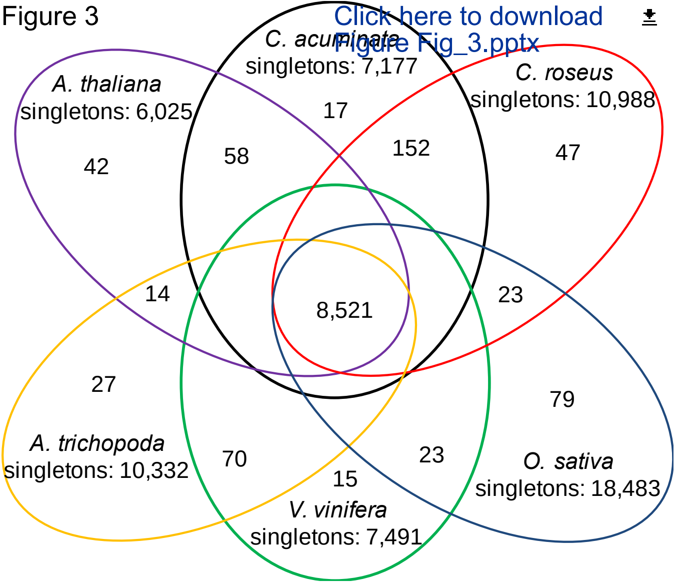

Figure 4

Click here to download Figure Fig_4_updated.pptx

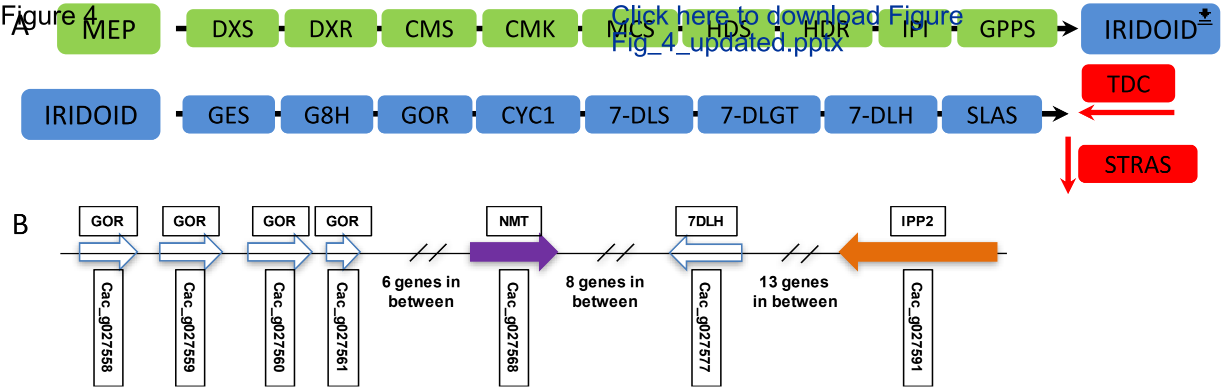

Supplementary Table 2

Click here to access/download
Supplementary Material
Supplemental_Table_2.xlsx

Supplementary Table 3

Click here to access/download
Supplementary Material
Supplemental_Table_3.xlsx

Supplementary Table 4

Click here to access/download
Supplementary Material
Supplemental_Table_4.xlsx
