# Supplementary material for: De novo genome assembly of Camptotheca acuminata, a natural source of the anti-cancer compound camptothecin
Source: Gigascience. 2017 Jul 24;6(9):1–7. doi: 10.1093/gigascience/gix065 (PMC5737489; doi:10.1093/gigascience/gix065)
Supplement: Additional file — Table S1: RNA-sequencing libraries used in this study. [file supplemental_table_1.docx]

**Additional file**

**Supplemental table:**

**Table S1. RNA-sequencing libraries used in this study.**

| BioProject ID | BioSample ID | Tissue | No. cleaned reads | Estimated bases |
| --- | --- | --- | --- | --- |
| PRJNA80029 | SAMN00255206 | mature leaf | 90,862,580 | 5,451,754,800 |
| PRJNA80029 | SAMN00255207 | immature bark | 84,537,958 | 5,072,277,480 |
| PRJNA80029 | SAMN00255208 | root | 88,940,668 | 5,336,440,080 |
| PRJNA80029 | SAMN00255215 | young flower | 71,435,806 | 4,286,148,360 |
| PRJNA80029 | SAMN00255216 | immature fruit | 84,250,338 | 5,055,020,280 |
| PRJNA80029 | SAMN00255217 | mature fruit | 47,811,342 | 2,868,680,520 |
| PRJNA80029 | SAMN00255222 | cotyledons | 74,037,722 | 4,442,263,320 |
| PRJNA80029 | SAMN00255223 | upper stem | 76,105,786 | 4,566,347,160 |
| PRJNA80029 | SAMN00255224 | lower stem | 72,680,940 | 4,360,856,400 |
| PRJNA361128 | SAMN06229771 | root | 55,435,804 | 7,224,198,331 |
| Total |  |  | 771,909,254 | 49,309,244,481 |
