# Supplementary material for: De novo genome assembly of Camptotheca acuminata, a natural source of the anti-cancer compound camptothecin
Source: Gigascience. 2017 Jul 24;6(9):1–7. doi: 10.1093/gigascience/gix065 (PMC5737489; doi:10.1093/gigascience/gix065)
Supplement: Response-to-Reviewer-Comments_Original-Submission.pdf [file gix065_response-to-reviewer-comments_original-submission.pdf]

Author's Response To Reviewer Comments

Dear Dr. Zauner

We appreciate the reviewers and your comments on our manuscript. We have revised the manuscript to address these concerns and hope our manuscript is now suitable for publication in GigaScience. We have provided a point-by-point response to your and the reviewer's comments below for your consideration.

Sincerely,
C. Robin Buell

Response to Reviewer's Comments

Please pay particular attention to the comments of reviewer 1 regarding missing or unclear methodological details.

Reviewer 1 also feels the specific examples in Fig. 4 & 5 are not necessary for a "data note" article and don't add much. I agree with the reviewer that the focus of the data note should be on genome wide patterns, but I do see potential value in your specific examples, in terms of demonstrating use cases for your data set.

On the other hand, the reviewer may be right that these examples (Fig 4 & 5) are quite preliminary. If you wish to keep the examples after reading the reviewer's report, I feel you need to make it clearer that the purpose is not to test specific biological hypotheses in detail, but to showcase potential use cases of the genome data, highlighting the preliminary nature of these analyses.

Author Response: While we do concur with the reviewer that the data as shown in Figure 4 and 5 are not genome-wide patterns, we felt that it was important to provide examples of use of the C. acuminata genome. To address these comments, we have removed Figure 5 and associated text completely from the manuscript. We have also clarified within the manuscript with additional text the rationale for including the analysis of the camptothecin biosynthetic pathway which refers to Figure 4. We have also moved the section on the camptothecin biosynthetic pathway to the section entitled "Uses for the C. acuminata genome sequence and annotation".

In terms of presenting methods, please note that we support and encourage the use of protocols.io (https://www.protocols.io/) and Research Ressource Identifiers (RRIDs, https://scicrunch.org/resources), where applicable.

Reviewer #1: This data note article describes the genome assembly, annotation, and RNA-Seq datasets, produced for the species Camptotheca acuminata, a source of the anti-cancer component camptothecin.
The assembly and annotation appear to be of satisfying quality. Few analyses were performed to

identify the camptothecin biosynthesis pathway and quantify tandem duplicated genes. As a data note, this article is not expected to contain numerous analyses, but it would be better to display only large scale (whole genome) analyses, rather than focusing on examples (as in figures 4 and 5), unless biologically relevant interpretations can be made out of those examples.

Below is a detailed list of comments:
Line 56: "and highlight the complexity of the gene complement in this species": how is the complexity of the gene complement highlighted in the article? Are the authors referring to the high number of tandem duplicated genes? If so, the sentence should be replaced by a more accurate statement.

Author Response: This sentence has been edited to more clearly state our results.

Line 67: how many transcripts were removed (best hits being a non-plant sequence)?

Author Response: The number of contaminants removed has been added to this sentence

Line 95: The % of Ns in the assembly should be stated, and in Table 2 as well (around 0.9%?).

Author Response: This has been added to the text and Table 2.

Line 109: "we use a novel genome annotation method". Could the authors be more explicit as to how it is "novel": AUGUSTUS and PASA are not novel, do they mean the combination of the two?

Author Response: This sentence has been edited to more clearly state our methods.

Line 134 and Figure 2B: the text mentions two mutations that confer resistance to camptothecin. The second mutation is in agreement with the species producing (S) or not producing (N) camptothecin, but the first mutation is not. For instance, "N" is found in all Ophiorrhiza species (producing camptothecin or not): can the authors comment on this?

Author Response: We edited the sentence to clarify these two mutations.

Orthology analysis (lines 158-160): gene ontology analyses are reported for "singleton" genes, but are there enriched terms among the genes that are not singletons, and especially among the ones that are in tandem ?

Author Response: We moved the section on the paralogous genes, including the tandem gene duplicates, to the Gene duplication and orthology subsection and performed GO enrichment tests.

Figure 3 is not easy to read: Venn diagrams should not be used for more than 4 samples. It would be better to choose another visualization (like UpSet).

Author Response: We investigated UpSet and while it appears to be a very good tool for

interactive data exploration, we don't think it is the best for Orthology analyses. Indeed, Venn diagrams are very standard for showing results from orthology analyses, as indicated by the multiple instances in Gigascience publications (listed below) that use Venn diagrams. Thus, we have retained Figure 3 as Venn diagram.

Xie et al., Gigascience (2017) 6 (5): 1-7.
DOI: https://doi.org/10.1093/gigascience/gix018

Kim et al., Gigascience (2017) 6 (3): 1-8.
DOI: https://doi.org/10.1093/gigascience/giw009

Kang et al., Gigascience (2017) 6 (1): 1-9.
DOI: https://doi.org/10.1093/gigascience/giw010

Peng et al., Gigascience (2016) 5 (1): 1-14.
DOI: https://doi.org/10.1186/s13742-016-0122-9

Calla et al., Gigascience (2015) 4 (1): 1-5.
DOI: https://doi.org/10.1186/s13742-015-0075-4

Line 168 and table 3: it is written that genes were identified "based on sequence identity and orthology with validated genes from C roseus", although in table 3 some genes have similarities with C camptotheca genes.

Are those genes the ones available from Genbank (described in line 135)? The MEP and iridoid pathway genes that were used to scan the C camptotheca assembly should be described in more detail. Also, table 3 title should be more explicit.

Author Response: We have clarified this text and provided a citation for the source of the query genes. To further improve the clarify we have limited the query genes in Table 3 to just C. roseus. We have edited the title of Table 3 to be more precise in its content.

Lines 174-186: this part, as figure 4, is very descriptive. What biological hypotheses can be made to explain the tandem amplification of GOR genes? What is the evolutionary scenario of duplications that could be proposed for this scaffold? As is, the figure should be removed or moved to the supplementary section.

Author Response: We have added new text and citations to explain the significance of gene duplication of the GOR genes. As described above, we feel Figure 4 is valuable to the reader, especially readers interested in camptothecin biosynthesis.

"Tandem duplicated genes and their differential expression":
The title of the section should be changed since no analysis of differential expression is described in the text.

Author Response: We have deleted this section yet retained the paralogous and tandem gene duplication results which are now in the section entitled “Gene duplication and orthology analyses”

Lines 203-205 and figure 5: the example of the late embryogenesis hydroxyprolin-rich glycoprotein is only descriptive and does not provide evidence of neo-functionalization. The figure should be removed. It would be more interesting to quantify, on the whole genome, how many tandem duplicated genes show differential expression (with an appropriate statistical method comparing expression profiles across tissues).

Author Response: This section has been deleted including the Figure.

Figures:
Figure3: data should be displayed otherwise (not as a Venn diagram)

Author Response: We feel Venn diagrams are an appropriate way to present orthology analyses as outlined above

Figure4: if it is not removed, the figure needs to be improved. It is not goodlooking at the moment (especially part A)

Author Response: We have revised this figure.

Reviewer #2: The manuscript presents a high quality genome assembly of C. acuminata, a tropical tree that produces a natural anti-cancer compound. It also represents the first genome from the Nyssaceae family.The genome assembly and annotation is of high quality, and several innovative methods were followed to ensure that the transcriptome assembly and annotations is stringent. The authors made use of the generated resource to identify candidate genes in the camptothecin biosynthesis pathway. This genomic resource is already producing valuable information on the production of these anti-cancer compounds.

The manuscript is of high quality, and written in a concise fashion. All the needed information regarding the assembly, annotation and data mining has been presented in a clear manner.

I recommend the acceptance of this manuscript without any additional revisions.

Author Response: We appreciate the positive comments by the reviewer.
