# Supplementary material for: De novo genome assembly of Camptotheca acuminata, a natural source of the anti-cancer compound camptothecin
Source: Gigascience. 2017 Jul 24;6(9):1–7. doi: 10.1093/gigascience/gix065 (PMC5737489; doi:10.1093/gigascience/gix065)
Supplement: Response-to-Reviewer-Comments_Revision-1.pdf [file gix065_response-to-reviewer-comments_revision-1.pdf]

## Author's Response To Reviewer Comments

Hans,

We have uploaded our revised manuscript to the Gigascience portal. To address the concerns of the reviewer on our Venn diagram of orthology, we redid the analyses with just four species and updated the Venn Diagram (Figure 3), Tables S2 and Table S3, and associated text to reflect the new set of orthologous/paralogous groups. We have also added additional datasets as requested to our Dryad DOI to provide the associated datasets for public release. We have made our SRA records public already and will release the C. acuminata genome through the Medicinal Plant Genomics Resource as soon as our manuscript is accepted. We hope that our manuscript and associated datasets are now suitable for publication in Gigascience.

Sincerely.
C. Robin Buell
