# Supplementary material for: De novo genome assembly of Camptotheca acuminata, a natural source of the anti-cancer compound camptothecin
Source: Gigascience. 2017 Jul 24;6(9):1–7. doi: 10.1093/gigascience/gix065 (PMC5737489; doi:10.1093/gigascience/gix065)
Supplement: Reviewer-1-Report-(Original-Submission).pdf [file gix065_reviewer-1-report-(original-submission).pdf]

Reviewer Report

Title: De novo genome assembly of Camptotheca acuminata, a natural source of the anti-cancer compound camptothecin

Version: Original Submission **Date:** 5/30/2017

Reviewer name: France Denoeud

Reviewer Comments to Author:

This data note article describes the genome assembly, annotation, and RNA-Seq datasets, produced for the species Camptotheca acuminata, a source of the anti-cancer component camptothecin.The assembly and annotation appear to be of satisfying quality. Few analyses were performed : to identify the camptothecin biosynthesis pathway and quantify tandem duplicated genes. As a data note, this article is not expected to contain numerous analyses, but it would be better to display only large scale (whole genome) analyses, rather than focusing on examples (as in figures 4 and 5), unless biologically relevant interpretations can be made out of those examples.Below is a detailed list of comments:Line 56 : "and highlight the complexity of the gene complement in this species" : how is the complexity of the gene complement highlighted in the article? Are the authors referring to the high number of tandem duplicated genes? If so, the sentence should be replaced by a more accurate statement.Line 67: how many transcripts were removed (best hits being a non-plant sequence) ?Line 95 : The % of Ns in the assembly should be stated, and in Table 2 as well (around 0.9% ?).Line 109: "we use a novel genome annotation method". Could the authors be more explicit as to how it is "novel" : AUGUSTUS and PASA are not novel, do they mean the combination of the two?Line 134 and Figure 2B: the text mentions two mutations that confer resistance to camptothecin. The second mutation is in agreement with the species producing (S) or not producing (N) camptothecin, but the first mutation is not. For instance "N" is found in all Ophiorrhiza species (producing camptothecin or not): can the authors comment on this?Orthology analysis (lines 158-160) : gene ontology analyses are reported for "singleton" genes, but are there enriched terms among the genes that are not singletons, and especially among the ones that are in tandem ? Figure 3 is not easy to read: Venn diagrams should not be used for more than 4 samples. It would be better to choose another visualization (like UpSet).Line 168 and table 3 : it is written that genes were identified "based on sequence identity and orthology with validated genes from C roseus", although in table 3 some genes have similarities with C camptotheca genes. Are those genes the ones available from Genbank (described in line 135) ? The MEP and iridoid pathway genes that were used to scan the C camptotheca assembly should be described in more detail. Also, table 3 title should be more explicit.Lines 174-186: this part, as figure 4, is very descriptive. What biological hypotheses can be made to explain the tandem amplification of GOR genes? What is the evolutionary scenario of duplications that could be proposed for this scaffold? As is, the figure should be removed or moved to the supplementary section."Tandem duplicated genes and their differential expression":The title of the section should be changed since no analysis of differential expression is described in the text. Lines 203-205 and figure 5 : the example of the late embryogenesis hydroxyprolin-rich glycoprotein is only descriptive and does not provide evidence of neo-functionalization. The figure should be removed. It

would be more interesting to quantify, on the whole genome, how many tandem duplicated genes show differential expression (with an appropriate statistical method comparing expression profiles across tissues).Figures:Figure3: data should be displayed otherwise (not as a Venn diagram)Figure4 : if it is not removed, the figure needs to be improved. It is not goodlooking at the moment (especially part A)

Level of Interest

Please indicate how interesting you found the manuscript: An article whose findings are important to those with closely related research interests

Quality of Written English

Please indicate the quality of language in the manuscript: Acceptable

Declaration of Competing Interests

Please complete a declaration of competing interests, considering the following questions:

- Have you in the past five years received reimbursements, fees, funding, or salary from an organisation that may in any way gain or lose financially from the publication of this manuscript, either now or in the future?
- Do you hold any stocks or shares in an organisation that may in any way gain or lose financially from the publication of this manuscript, either now or in the future?
- Do you hold or are you currently applying for any patents relating to the content of the manuscript?
- Have you received reimbursements, fees, funding, or salary from an organization that holds or has applied for patents relating to the content of the manuscript?
- Do you have any other financial competing interests?
- Do you have any non-financial competing interests in relation to this paper?

If you can answer no to all of the above, write 'I declare that I have no competing interests' below. If your reply is yes to any, please give details below.

I declare that I have no competing interests

I agree to the open peer review policy of the journal. I understand that my name will be included on my report to the authors and, if the manuscript is accepted for publication, my named report including any attachments I upload will be posted on the website along with the authors' responses. I agree for my report to be made available under an Open Access Creative Commons CC-BY license (http://creativecommons.org/licenses/by/4.0/). I understand that any comments which I do not wish to be included in my named report can be included as confidential comments to the editors, which will not be published.

I agree to the open peer review policy of the journal
