# Supplementary material for: De novo genome assembly of Camptotheca acuminata, a natural source of the anti-cancer compound camptothecin
Source: Gigascience. 2017 Jul 24;6(9):1–7. doi: 10.1093/gigascience/gix065 (PMC5737489; doi:10.1093/gigascience/gix065)
Supplement: Reviewer-2-Report-(Original-Submission).pdf [file gix065_reviewer-2-report-(original-submission).pdf]

Reviewer Report

Title: De novo genome assembly of Camptotheca acuminata, a natural source of the anti-cancer compound camptothecin

Version: Original Submission **Date:** 6/7/2017

Reviewer name: Charles Hefer

Reviewer Comments to Author:

The manuscript presents a high quality genome assembly of C. acuminata, a tropical tree that produces a natural anti-cancer compound. It also represents the first genome from the Nyssaceae family.The genome assembly and annotation is of high quality, and several innovative methods were followed to ensure that the transcriptome assembly and annotations is stringent. The authors made use of the generated resource to identify candidate genes in the camptothecin biosynthesis pathway. This genomic resource is already producing valuable information on the production of these anti-cancer compounds.The manuscript is of high quality, and written in a concise fashion. All the needed information regarding the assembly, annotation and data mining has been presented in a clear manner.I recommend the acceptance of this manuscript without any additional revisions.

Level of Interest

Please indicate how interesting you found the manuscript: An article whose findings are important to those with closely related research interests

Quality of Written English

Please indicate the quality of language in the manuscript: Acceptable

Declaration of Competing Interests

Please complete a declaration of competing interests, considering the following questions:

- Have you in the past five years received reimbursements, fees, funding, or salary from an organisation that may in any way gain or lose financially from the publication of this manuscript, either now or in the future?
- Do you hold any stocks or shares in an organisation that may in any way gain or lose financially from the publication of this manuscript, either now or in the future?
- Do you hold or are you currently applying for any patents relating to the content of the manuscript?
- Have you received reimbursements, fees, funding, or salary from an organization that holds or has applied for patents relating to the content of the manuscript?
- Do you have any other financial competing interests?

- Do you have any non-financial competing interests in relation to this paper?

If you can answer no to all of the above, write 'I declare that I have no competing interests' below. If your reply is yes to any, please give details below.

I declare that I have no competing interests

I agree to the open peer review policy of the journal. I understand that my name will be included on my report to the authors and, if the manuscript is accepted for publication, my named report including any attachments I upload will be posted on the website along with the authors' responses. I agree for my report to be made available under an Open Access Creative Commons CC-BY license (http://creativecommons.org/licenses/by/4.0/). I understand that any comments which I do not wish to be included in my named report can be included as confidential comments to the editors, which will not be published.

I agree to the open peer review policy of the journal
